# Vitamin D attenuates sphingosine-1-phosphate (S1P)-mediated inhibition of extravillous trophoblast migration

**DOI:** 10.1016/j.placenta.2017.09.009

**Published:** 2017-12

**Authors:** Melissa Westwood, Khiria Al-Saghir, Sarah Finn-Sell, Cherlyn Tan, Elizabeth Cowley, Stéphane Berneau, Daman Adlam, Edward D. Johnstone

**Affiliations:** a Maternal and Fetal Health Research Centre, Division of Developmental Biology & Medicine, School of Medical Sciences, Faculty of Biology, Medicine and Health, University of Manchester, Manchester Academic Health Sciences Centre, Manchester, UK; b Maternal and Fetal Health Research Centre, St Mary's Hospital, Central Manchester Universities NHS Foundation Trust, Manchester Academic Health Sciences Centre, Manchester, UK

**Keywords:** S1P, Vitamin D, GPCR, Trophoblast, Migration, Lipid signalling, Placenta, Pre-eclampsia

## Abstract

**Introduction:**

Failure of trophoblast invasion and remodelling of maternal blood vessels leads to the pregnancy complication pre-eclampsia (PE). In other systems, the sphingolipid, sphingosine-1-phosphate (S1P), controls cell migration therefore this study determined its effect on extravillous trophoblast (EVT) function.

**Methods:**

A transwell migration system was used to assess the behaviour of three trophoblast cell lines, Swan-71, SGHPL-4, and JEG3, and primary human trophoblasts in the presence or absence of S1P, S1P pathway inhibitors and 1,25(OH)_2_D_3_. QPCR and immunolocalisation were used to demonstrate EVT S1P receptor expression.

**Results:**

EVTs express S1P receptors 1, 2 and 3. S1P inhibited EVT migration. This effect was abolished in the presence of the specific S1PR2 inhibitor, JTE-013 (p < 0.05 versus S1P alone) whereas treatment with the S1R1/3 inhibitor, FTY720, had no effect. In other cell types S1PR2 is regulated by vitamin D; here we found that treatment with 1,25(OH)_2_D_3_ for 48 or 72 h reduces S1PR2 (4-fold; <0.05), but not R1 and R3, expression. Moreover, S1P did not inhibit the migration of cells exposed to 1,25(OH)_2_D_3_ (p < 0.05).

**Discussion:**

This study demonstrates that although EVT express three S1P receptor isoforms, S1P predominantly signals through S1PR2/Gα_12/13_ to activate Rho and thereby acts as potent inhibitor of EVT migration. Importantly, expression of S1PR2, and therefore S1P function, can be down-regulated by vitamin D. Our data suggest that vitamin D deficiency, which is known to be associated with PE, may contribute to the impaired trophoblast migration that underlies this condition.

## Highlights

• Sphingosine-1-Phosphate (S1P) controls extravillous trophoblast (EVT) migration.
• Control of migration is exerted through Sphingosine-1-Phosphate receptor 2 (S1PR2).
• Vitamin D attenuates the expression of S1PR2 and therefore promotes EVT migration in the presence of S1P.

## 1 Introduction

Normal placental development depends on the ability of extravillous trophoblast (EVT) to colonise and transform the spiral arteries of the maternal uterus into passive conduits that are capable of supplying the placenta with sufficient oxygen- and nutrient-rich maternal blood to meet the increasing metabolic demands of the growing fetus [1]. Abnormalities in EVT function result in the shallow placental invasion seen in the serious pregnancy disease pre-eclampsia (PE) [2]. Options for treating PE are currently extremely limited [3] so increased understanding of the molecular basis of EVT migration and spiral artery remodelling is needed in the search for medical therapies for this pregnancy disease.

During normal spiral artery remodelling, EVT reach the vessel wall by migrating through the lumen or decidual stroma. Oxygen, cytokines and ECM are known to influence this process [4], [5], [6] but the molecular mechanisms involved in integrating the signals derived from these environmental cues are unknown. Data from studies of other vascular beds point to a crucial role for the bioactive sphingolipid metabolite, sphingosine-1-phosphate (S1P) [7], [8], [9]. Moreover, a study using metabolomics to profile placental tissue in pregnancies complicated by PE [10] identified deranged sphingolipid metabolism as one of only a handful of pathways altered in this condition.

**Fig. 1:**
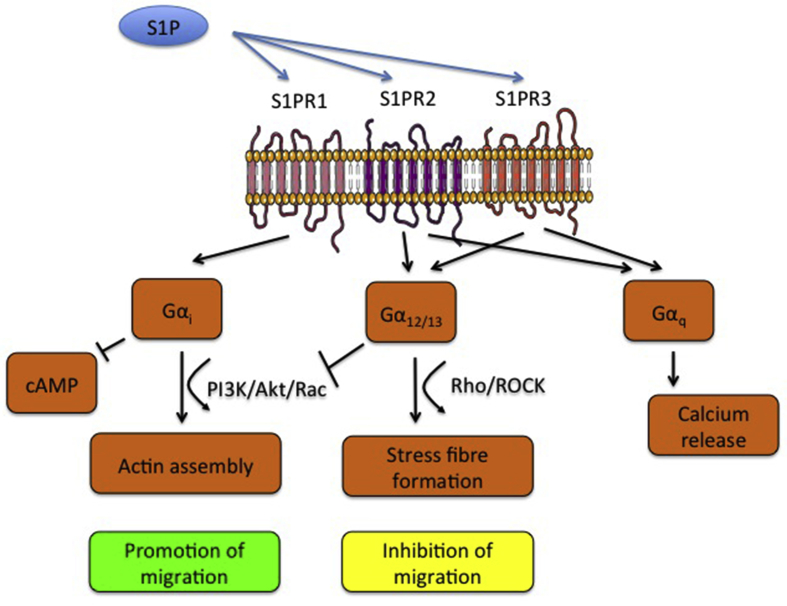
S1PR1-3 G-protein linked signalling cascades.

S1P activates a family of G-protein coupled receptors (S1PR1-5), whose expression varies with cell type [11]. Each receptor links to different G-proteins allowing for a variety of signalling pathways to be activated [12]. S1PR1 links exclusively to Gα_i_ [13] and can therefore inhibit adenylate cyclase and the formation of cAMP [14], [15] yet stimulate ERKs and PI3K [16]. S1PR2 couples to Gα_i_, but also to Gα_q_, which enables activation of phospholipase C and calcium mobilisation [15], and Gα_12/13_, which activates Rho and actin cytoskeleton reassembly (Fig. 1) [17]. S1PR3 activates the same downstream signalling molecules as S1PR2 [18]. Consequently, the net cellular response depends on receptor repertoire and S1P can have stimulatory or inhibitory effects on cell migration depending on whether S1PR1/3 or S1PR2 is activated [17]. This study aimed to characterise the S1P receptor repertoire and functional response of EVT to determine whether deranged S1P signalling could contribute to abnormal placentation caused by deficient EVT migration. Following our discovery that S1P inhibited trophoblast migration in an S1PR2-dependent manner, we identified vitamin D, known to be deficient in pre-eclampsia [19] as a regulator of S1PR2 and trophoblast function in early placental development.

## 2 Materials and methods

### 2.1 Tissue and cell culture

Early first trimester (6–9 week) human placental tissue was collected following elective surgical or medical termination of pregnancy with maternal informed consent and appropriate ethical review (NREC 08/H1010/28). The human first trimester EVT cell line Swan-71 [20] was maintained in 1:1 DMEM:Ham's F-12 (Lonza, Cambridge, UK) supplemented with 10% fetal calf serum (FCS; Biosera, Maidenhead, UK), penicillin (0.1U/ml), streptomycin (100 μg/ml) and amphotericin (250 ng/ml; Gibco, Paisley, UK) at 37C, 5% CO_2_. SGHPL-4 cells [21], [22] were cultured in Ham's F-10 (Lonza) with 10% FCS and antibiotic/antimycotic supplements (as described above) at 37 °C, 5% CO_2_. JEG3 cells (European Collection of Authenticated Cell Cultures 92120308) were cultured in MEM with 10% FCS, 1% non-essential amino acids, 2 mM glutamine and antibiotic/antimycotic supplements at 37C, 5% CO_2_.

### 2.2 Immunostaining

Tissue was fixed in 4% paraformaldehyde then embedded in paraffin and cut into 5  μM sections. These were boiled in 0.01M sodium citrate buffer, pH 6.0 for antigen retrieval then incubated with 3% hydrogen peroxide for 10 min followed by 5% bovine serum albumin (BSA) for 30 min. Rabbit non-immune IgG (Dako, Ely, UK), mouse non-immune IgG (Dako) or primary antibodies (rabbit anti-S1PR1 (1:50; #sc-25489), anti-S1PR2 (1:100; #sc-25491), S1PR3 (1:100; #sc-30024), all Santa Cruz Biotechnology, or mouse anti-HLA-G (1:50; #7759, Abcam, Cambridgeshire, UK) were applied for 2 h at room temperature, then sections were incubated with biotinylated goat anti-rabbit IgG antibody (1:300; Dako) or goat anti-mouse IgG antibody (1:300, Dako) for 30 min followed by avidin peroxidase (5 μg/ml) for 45 min. Immune complexes were visualised using diaminobenzidine then sections were counterstained with Harris's hematoxylin. Sections were observed with an Olympus BX41 microscope (Olympus, UK;×40 objective) and photographed using a QICAM fast 1364 digital camera (QImaging, UK). Cells were methanol-fixed then incubated with 0.25% Triton X-100 for 5 min before blocking (10% donkey serum) for 30 min. Rabbit or mouse non-immune IgG or primary antibodies (as above) were applied overnight at 4 °C and then cells were incubated for 1 h at room temperature with an Alexa Fluor 488-conjugated donkey anti-rabbit IgG (Life Technologies, Paisley, UK) or donkey anti-mouse IgG (Life Technologies) secondary antibody. Nuclei were stained with 4′,6-diamidino-2-phenylindole (DAPI; Vector, Cambridge, UK). Cells were viewed and imaged using an Olympus IX70 microscope and camera (×40 objective).

### 2.3 EVT cell line migration assay

Cells were seeded into transwell filters (8  μM pores; BD Biosciences) and incubated for 24 h in media (as above except FCS was reduced to 1%) containing 0.1–10 μM S1P (Sigma-Aldrich, Poole, UK) or vehicle (methanol). Cells were methanol-fixed then those on the upper surface of the membrane were removed using a cotton swab and the remainder were stained with Harris's hematoxylin (Sigma). Cells were viewed by light microscopy (Olympus BX41 microscope,×10 magnification); the number of cells in 6 fields of view were counted. In some experiments, the S1PR-1/3 inhibitor, FTY720 or pFTY720 (100 nM, Cayman Chemical Company, Ann Arbor, USA), the S1PR2 inhibitor, JTE-013 (100 nM, Tocris, Abingdon, UK) or the Rho kinase inhibitor, Y27632 (10 μM; Cytoskeleton Inc., USA) was added to cultures for 30 min before treatment with 100 nM S1P for a further 24 h. In experiments to assess the affect of 1,25-dihydroxyvitamin D (D_3_; Sigma) on EVT migration, D_3_ (1-10 nM) was added for 48 h or 72 h before incubation with 100 nM S1P for a further 24 h.

### 2.4 Primary EVT migration assay

Terminal portions of villi from first trimester placentas were seeded onto collagen gels and cultured overnight in a 1:1 mix of DMEM and Ham's F12 containing l-glutamine (2 mmol/L), penicillin (100 IU/ml), and streptomycin (100 μg/ml) at 37 °C in a humidified incubator (5% CO2). Those that had attached (radial stress lines in the collagen gel) and showed evidence of functioning extravillous trophoblast (presence of cell outgrowth from the explant border) were selected for further experimentation and imaged (using a Nikon Eclipse TE 200 inverted microscope and a Nikon Coolpix 990 digital camera (Nikon, Tokyo, Japan)) at this time point (deemed 0 h) explants were then cultured with S1P (100 nM) or fetal calf serum (control) for 24 h before reimaging allowing changes in outgrowth and migration to be assessed.

### 2.5 cAMP analysis

Swan-71 cells were stimulated with forskolin (5  μM; Sigma) in the absence or presence of an S1PR1/3 (100 nM) and/or R2 inhibitor (100 nM) for 30 min before the addition of S1P (100 nM) or vehicle for 30 min. Levels of cAMP were determined using the cAMP-Glo assay in accordance with the manufacturer's instructions (Promega, Southampton, UK).

### 2.6 Western blotting

Lysates of Swan-71 cells were prepared in RIPA buffer and 50 μg protein from each sample was resolved by SDS-PAGE and transferred to nitrocellulose membranes for Western blotting with a rabbit antibody specific for phosphorylated Akt (1:1000; #9271, Cell-Signalling Technology). Immune complexes were visualised by probing membranes with a HRP-conjugated anti-rabbit IgG antibody (1:2000; Dako) followed by chemiluminescence reagents and exposure to X-ray film. Membranes were stripped (2% SDS, 100 mM β-mercaptoethanol, 50 mM Tris, pH 6.8, for 30 min at 50 °C) and re-probed with an antibody that recognises total Akt (1:1000; #4685, Cell Signalling Technology) in order to confirm equal protein loading.

### 2.7 Analysis of the actin cytoskeleton

Swan-71 cells were treated with vehicle, S1P (100 nM), or S1P and the Rho kinase inhibitor (Y-27632; 10 μM) for 30 min then fixed in 4% formaldehyde, 1% triton for 5 min. After washing, cells were stained using Alexa Fluor 594-conjugated phalloidin (150 nM; Life Technologies, UK) in 1% BSA for 40 min; nuclei were stained with DAPI. Cells were observed and photographed using an Axio Observer.Z1 microscope (Carl Zeiss Ltd, UK;×40 objective) and an Axiocam MR microscope camera (Carl Zeiss).

### 2.8 Rac activity assay

The effect of S1P on the activity of Rac in Swan-71 cells was analysed using the G-LISA Rac Activation Biochem Kit in accordance with the manufacturer's instructions (Cytoskeleton, Inc, USA). Briefly, Swan-71 cells were cultured in the absence or presence of 100 nm S1P or 100 ng/ml EGF (as a positive control) for 60 s, lysed and then the level of active Rac in 0.5 mg/ml protein from each sample was determined using an antibody to capture GTP-bound Rac followed sequentially, by a Rac specific antibody, a HRP-labelled antibody, HRP detection reagent and finally, measurement of absorbance at 490 nM.

### 2.9 QPCR

Total RNA; Absolutely RNA microprep kit (Stratagene, USA), reversed transcribed using an Affinity Script Multi Temperature Kit (Stratagene). QPCR was conducted using Brilliant SYBR Green qPCR Master Mix (Stratagene), 5-carboxy-x-rhodamine reference dye and specific primers (200 nM; Invitrogen) for *S1PR1-3*, *VDR* and the housekeeping gene, *YWHAZ* (Table 1) in a Stratagene Mx3000P Real Time PCR machine. mRNA levels were quantified against standard curves generated from human reference total RNA.

**Table 1:** Sequence of primer pairs used for qPCR analysis. *S1PR1* was amplified using an annealing temperature of 57C; all other reactions utilised an annealing temperature of 60C.

Gene	Primer Sequence
S1PR1	F: ACCCCATCATTTACACTCTGACC R:GGTTGTCCCCTTCGTCTTTCT
S1PR2	F: GCCACTTGACGACTTCTC R: CTGAGCAATGAGGTCTGAG
S1PR3	F: CAGTGTGGATGTCTCTTACAG R: AGCAGAAGGCAGTGGATG
VDR	F: CTTCAGGCGAAGCATGAAGC R: CCTTCATCATGCCGATGTCC
YWHAZ	F: CCTGCATGAAGTCTGTAACTGAG R:TTGAGACGACCCTCCAAGATG

### 2.10 Statistical analysis

Data are presented as mean ± standard error of the mean (SEM) or median. Data were analysed using a one-way analysis of variance (Kruskal-Wallis with Dunn's post-hoc test) or Wilcoxon signed-rank test; P < 0.05 was considered significant. Statistical analyses were performed using Graph Pad Prism (6.0).

## 3 Results

### 3.1 EVT express S1P receptors 1-3

**Fig. 2:**
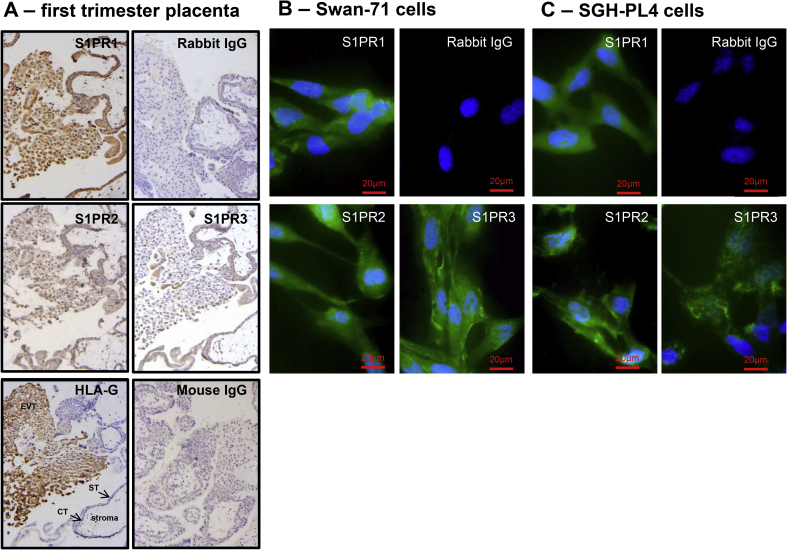
Expression of S1P receptors in primary extravillous trophoblast (EVT) and EVT cell lines. S1PR protein localisation was determined by immunostaining of human first trimester placenta **(A)**, Swan-71 cells **(B)** and SGHPL-4 cells **(C)** counterstained with hematoxylin or DAPI. Staining for HLA-G was used to indicate extravillous trophoblast and some slides were incubated with IgG instead of primary antibody as a negative control. Bar represents 20 μM. Images are representative of 4 independent experiments.

Immunohistochemical analysis revealed that in primary tissue, the migratory trophoblast in villous cell columns (identified by staining for HLA-G in serial sections) express S1PR1, R2 and R3, though predominantly R1 and R2 (Fig. 2A). All three receptors were expressed by syncytio- and cytotrophoblast and also cells in the stroma. S1PR1-3 protein was also detected in the EVT cell lines, Swan-71 (Fig. 2B) and SGHPL-4 cells (Fig. 2C).

### 3.2 S1P attenuates EVT migration via S1PR2

**Fig. 3:**
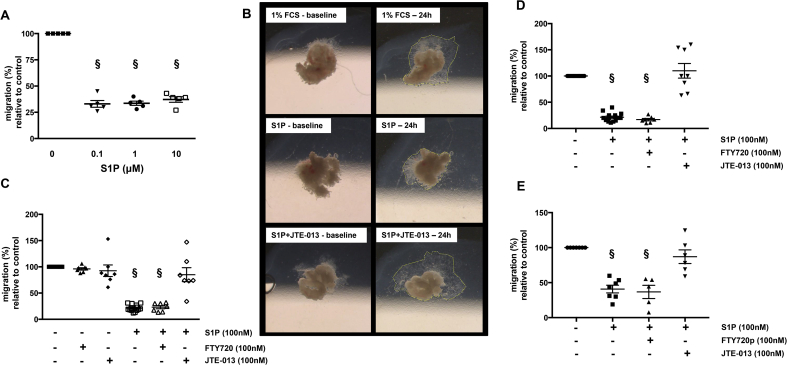
S1P attenuates EVT migration via S1PR2. **(A)** Swan-71 cells were seeded on cell culture transwell inserts, incubated for 24 h with varying concentrations of S1P (100nM-10μM) or vehicle control (methanol) and then the number of cells that had migrated through the filter (8 μM pores) was determined by counting the number of cells present in micrographs taken, using an Olympus inverted microscope at 10× magnification, of six different fields for each insert. Data are presented as percentage of cells, relative to control, that had migrated. Bar – mean ± SEM; § - p < 0.05; n = 5. **(B)** Primary placental villous tips were embedded in collagen, cultured for 24 h and then photographed (baseline). Tissue was treated with media containing either 1% fetal calf serum (control), 100 nM S1P or 100 nM S1P and 100 nM JTE013 for a further 24 h before photographing the same area of explant. The images shown are representative of those obtained from four independent experiments. Swan-71 cells **(C)**, SGHPL-4 cells **(D)** or JEG3 cells **(E)** were incubated with 100 nM of S1PR1/3 inhibitor, FTY720 (**C-D**) or FTY720p (**E**) or the S1PR2 inhibitor, JTE-013 (100 nM; **C-E**) for 30 min before the addition of vehicle or S1P (100 nM) and culture for 24 h. The number of cells that had migrated through the filter is shown as percentage relative to untreated cells. Bar – mean ± SEM; § - p < 0.05 versus untreated cells; n = 7.

S1P had no affect on the proliferation or viability of the EVT cell lines (data not shown) however, migration was significantly attenuated following exposure to S1P. 100 nM S1P was sufficient to cause a ∼3-fold reduction (to 33 (±3.2)%; p < 0.05) in Swan-71 cell migration (Fig. 3A). This dose had a similar effect on the motility of SGHPL-4 cells (Fig. 3D) and choriocarcinoma-derived JEG3 cells (Fig. 3E), which are recommended for use in studies of cell motility [23] (24 (±2.1)% and 40 (±5.5)% versus control respectively; p < 0.05). Incubation of human first trimester placental explants with 100 nM S1P also inhibited the migration of EVT (Fig. 3B), therefore all subsequent experiments were conducted using 100 nM S1P.

As trophoblast cells express three of the S1P receptor isoforms, we used inhibitors specific for R1/3 (FTY720 and pFTY720) and R2 (JTE-013) to determine which receptor was responsible for mediating S1P's effect on migration. Fig. 3C-E demonstrates that pre-treatment of Swan-71 (Fig. 3C), SGHPL-4 (Fig. 3D) and JEG3 (Fig. 3E) cells with JTE-013, but not FTY720 or pFTY720 abolished S1P's ability to inhibit migration, suggesting that S1P is signalling through S1PR2.

**Fig. 4:**
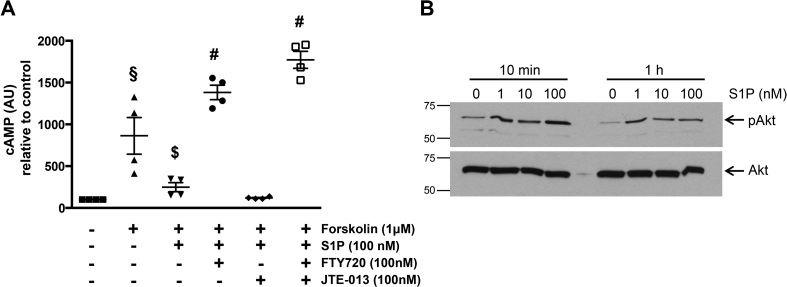
S1PR1 is active in EVT cells. **(A)** Swan-71 cells were cultured in the absence or presence of forskolin (1 μM) for 15 min. The S1PR2 inhibitor JTE-013 (100 nM), the S1P R1/3 inhibitor FTY720 (100 nM) or vehicle was added for 20 min then cells were cultured in the absence or presence of 100 nM S1P for a further 30 min before measuring intracellular cAMP levels. Data are from four independent experiments and are expressed relative to control. Bar – mean ± SEM; § - p < 0.05 versus untreated cells; $ - p < 0.05 versus forskolin treatment alone; # - p < 0.05 versus forskolin and S1P treatment. **(B)** Swan-71 cells were exposed to S1P (0-100 nM) for 10 min or 1 h then cell lysates were analysed by western blotting using antibodies that recognise phosphorylated (pAkT) or total Akt. Blots are representative of data obtained from 3 independent experiments.

**Fig. 5:**
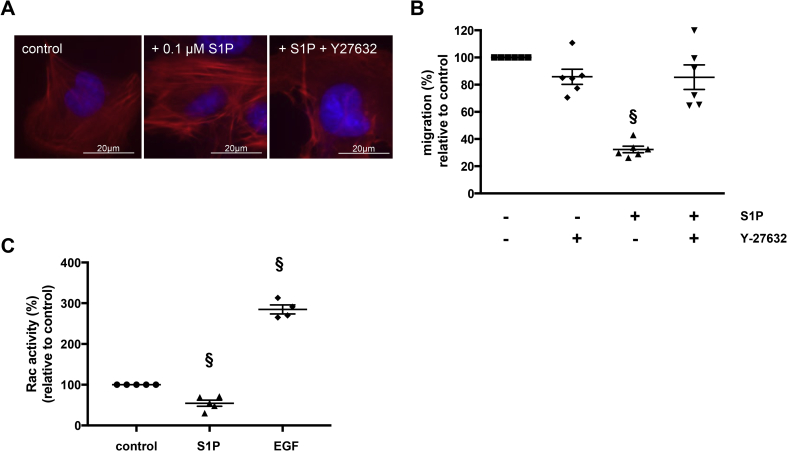
S1P activates RhoA/ROCK but inhibits Rac. Swan-71 cells were treated with vehicle (control), S1P (100 nM) or the Rho kinase inhibitor Y-27632 (10 μM) for 30 min followed by S1P (100 nM) for a further 30 min or 24 h before staining with Alexa Fluor 594-conjugated phalloidin and DAPI **(A)** or assessing migration **(B)** respectively. Bar – mean ± SEM; n = 6; § - p < 0.05 relative to culture in the absence of S1P. **(C)** Swan-71 cells were cultured in the absence (control) or presence of S1P (100 nM) or a known stimulator of cAMP synthesis, EGF (100 ng/ml), for 60 s, lysed and then the level of active Rac in 0.5 mg/ml protein from each sample was determined using the G-LISA Rac Activation Biochem Kit. Bar – mean ± SEM; n = 5; § - p < 0.05 relative to control.

We next investigated whether S1PR1, the other receptor isoform predominantly expressed by primary EVT, is active in Swan-71 cells by examining the effect of S1P on cAMP production. Forskolin-stimulated cAMP production was inhibited by S1P (Fig. 4A) and importantly, this effect was prevented by inhibiting S1PR1 and not R2 activity, suggesting that S1P inhibition of adenylate cyclase activity is mediated via S1PR1/Gα_i_ coupling (Fig. 4A). Further evidence for the ability of S1P to activate Gα_i_ was sought by investigating the activation status of the signalling molecule, Akt, which is known to be downstream of this G-protein [24]. We were also interested in determining whether activation of Gα_i_/Akt was S1P dose dependent as previous studies have suggested that this pathway might dominate at lower doses. Fig. 4B shows that S1P is capable of activating Akt and, importantly, that activation was apparent at all of the S1P doses used, confirming that S1P signalling through Gα_i_ is possible in EVT. Together, these data suggest that S1P/S1PR2 binding causes preferential activation of the Gα_12/13_/Rho/ROCK pathway rather than Gα_i_/Rac signalling, which is consistent with S1P's inhibitory affect on the migration of these cells. Indeed pre-treatment of Swan-71 cells with the ROCK inhibitor, Y27632, attenuated S1P-induced stress fibre formation (Fig. 5A) and S1P-inhibition of migration (Fig. 5B). Furthermore, treatment of Swan-71 cells with 100 nM S1P for 60 s caused a 50% decrease in activity (p < 0.05) of the pro-migratory GTPase, Rac (Fig. 5C).

### 3.3 S1P's inhibitory actions can be attenuated by vitamin D

**Fig. 6:**
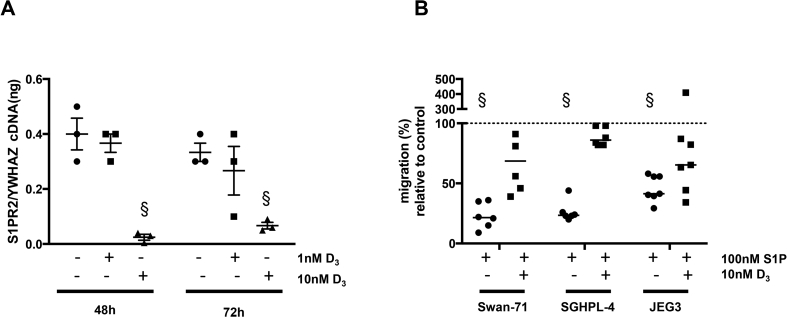
Vitamin D suppresses S1PR2 expression and attenuates S1P-mediated inhibition of EVT cell migration. Swan-71 cells were incubated with vehicle or 1-10 nM 1,25-dihydroxyvitamin D (D_3_) for 48 or 72 h then **(A)** the level of S1PR2 mRNA, normalised to the housekeeping gene YWHAZ, was measured by qPCR (n = 3). **(B)** Swan-71, SGHPL-4 or JEG3 cells were cultured in 10 nM D_3_ for 48 h then seeded in transwells and incubated in the absence or presence of 100 nM S1P for a further 24 h before assessing migration (n = 5-7). Bar – median; § - p < 0.05 versus cells cultured in the relevant control medium (absence or presence of D_3_), normalised to 100%.

The data described above suggest that reducing the expression of S1PR2 might attenuate S1P's inhibitory affect on EVT cell migration. We therefore investigated the effect of vitamin D on S1PR2 expression and the migratory ability of EVT as a study by Kikuta et al. demonstrated that vitamin D reduced S1PR2 expression on osteoclast precursor monocyte cells, both *in vitro* and *in vivo*, with a consequent increase in the migration of cells towards S1P [25]. We first confirmed that the trophoblast cell lines express the vitamin D receptor (data not shown) then cells were incubated with 1 or 10 nM of 1,25-dihydroxyvitamin D (D_3_) for either 48 h or 72 h. qPCR analysis revealed a significant reduction in the expression of S1PR2 mRNA following treatment with 10 nM D_3_ for either time period (Fig. 6A). Importantly, pre-treating cells with 10 nM D_3_ for 48 h or 72 h reversed the inhibitory effect of S1P on trophoblast cell migration (Fig. 6B).

## 4 Discussion

Although S1P is well known as a regulator of cellular motility [26], this is the first study to examine the effects of this bioactive lipid on the behaviour of human EVT.

Extracellular S1P functions through its receptors, S1PR1–5 and in this study we demonstrated the presence of S1PR1-3 on primary EVT cells and in our trophoblast models. Previous studies of other cell types have indicated that the effect of S1P on migration and invasion depends on which S1P receptor(s) are activated. We found that S1P, at doses relevant to circulating levels (0.4-1 μM range [27]), inhibited migration in our three EVT models, suggesting preferential activation of S1PR2. A previous study using the cell line HTR8/SVneo found that S1P promoted trophoblast cell migration [28], though these authors employed a lower concentration of S1P (10 nM) and it is possible that these cells, which are reported to have fewer transcripts in common with primary EVT than JEG3 cells [23], predominantly express S1PR1/3. Furthermore, it has recently been demonstrated that the HTR8/SVneo cell line contains a mixed population of different cell types, which may have resulted in non-EVT specific responses to S1P [29]. In order to substantiate the notion that S1PR2 is involved in inhibiting the migration of the trophoblast cell lines utilised on the current study, we tested the effects of the S1P analogue, FTY720, which functionally antagonises S1PR1/3, and JTE-013, the S1PR2 receptor antagonist on S1P actions in EVT; only the latter was able to abolish the inhibitory effect of S1P on trophoblast migration. This effect was independent of changes in cell number. S1PR2 can couple to Gα_12/13_, which activates RhoA and downstream molecules that ultimately lead to stress fibre formation and reduced motility, or Gα_i_, which links to another small GTPase, Rac that regulates the cellular cytoskeleton to promote cell migration. The functional data obtained in this study indicate that EVT, S1PR2 primarily couples to Gα_12/13_/RhoA, despite the demonstrable presence of active Gα_i_. This observation is in keeping with data from cells engineered to over-express S1PR2 in which S1P-mediated activation of ERK (a downstream marker of coupling to Gα_i_) was less efficient than in cells expressing S1PR1 or R3 [30].

RhoA mediates stress fibre formation and focal adhesion through a phosphorylation cascade beginning with Rho kinase (ROCK) to impede the activity of cofilin and myosin light-chain phosphatase and stabilise actin filaments. Cells with strong stress fibres are generally less motile and stress fibres are known to inhibit cell motility. Treatment of Swan-71 cells with S1P resulted in the induction of stress fibres which was blocked by pre-treatment with the specific Rho kinase inhibitor Y-27632. These findings are in agreement with those of Lepley et al., who found that S1P mediated formation of stress fibres and the inhibition of glioblastoma cell migration was dependent on S1PR2/Rho/ROCK [31], although studies of CHO cells that over-express S1PR2 suggest that Rho-kinase-independent mechanisms for S1PR2/Gα_12/13_/Rho regulating cell migration [17] also exist.

In addition to inhibiting cell migration by activating Rho and consequently ROCK, S1P-activated Rho may also attenuate cell motility by inhibiting the activity of the pro-migratory GTPase Rac (a downstream mediator of Gα_i_) [17], [32], [33] through mechanisms involving stimulation of a GTPase-activating protein (GAP) for Rac [34]. It is possible that in EVT robust activation of Gα_12/13_ following S1P/S1PR2 interactions masks Gα_i_-mediated stimulation of Rac, thus strategies aimed at enhancing the latter pathway, for example overexpression of Gα_i_, as demonstrated by Sugimoto et al. [17], or inhibition/down-regulation of GAPs, might overcome the consequences of S1PR2/Gα_12/13_ signalling for cell migration.

We found no difference in mRNA expression levels of S1PR1, R2 and R3 in our trophoblast cell models. S1P is reported to have a similar affinity for each of the S1P receptors R1-R3 [35], [36] and we demonstrated that S1PR1 was capable of responding to the concentration of S1P used by assessing the effect of S1P on cAMP production in the presence of the S1PR1 inhibitor FTY720. Nonetheless, S1P inhibited the migration of all of the trophoblast models investigated, which suggests that S1P preferentially activates signalling through S1PR2. It is unknown why this occurs in EVT and other cell types, but it is possible that the S1P receptors are subject to “biased signalling” in which specific receptor conformations, and the consequent signalling behaviours, are stabilised at the exclusion of other states.

This is the first study to examine the effect of vitamin D on S1P receptors in trophoblast. Vitamin D insufficiency/deficiency during pregnancy has been linked to an increased risk of developing preeclampsia [19]. Our key finding is that S1P's inhibitory affect on trophoblast migration was completely abolished by pre-treatment of cells with D_3_ and that this was mediated by down regulation of S1PR2 mRNA. These observations support those of Kikuta et al. [25] who found that in both *in vitro* and *in vivo* models, D_3_ suppressed the expression of S1PR2 and consequently alleviated S1P inhibition of osteoclast precursor monocyte migration. Interestingly Vitamin D and its analogue, BXL-628, have been shown to inhibit the migration of human and rat bladder smooth muscle cells by blocking the activation of the RhoA/ROCK signalling pathway [37]. The authors of this study did not investigate the molecules upstream of RhoA, however it is tempting to speculate that the effects of vitamin D were due to down-regulation of S1PR2 expression.

Observational studies in humans suggest that low D_3_ levels are associated with a number of pregnancy complications, including pre-eclampsia [38]. In the UK, all pregnant women are advised to supplement their diet by taking 400 international units (IU) vitamin D daily to counter the well-recognised bone defects associated with deficiency [39]. Such a dose is reported to raise circulating levels of the D_3_ precursor, 25-hydroxyvitamin D (25(OH)D), by 4 ng/ml (10 nM) [40]; it is likely that 1,25D levels are similarly increased following vitamin D supplementation which, according to the results of our study, would be sufficient to affect the expression of S1PR2. It is therefore interesting to note the findings of a recent systematic review which suggest that daily supplementation with 800-1000IU vitamin D protects against low birth weight [41]. Moreover, an analysis of over 23,000 nulliparous women taking part in the Norwegian Mother and Child Cohort study revealed that supplementary intake of vitamin D conferred a protective, albeit small, effect against developing pre-eclampsia and interestingly, supplementation in both early and late pregnancy was advantageous [42]. The authors suggest that in early pregnancy, vitamin D may be an important regulator of genes requires for successful pregnancy whereas later in gestation, it may influence the maternal immune response to the fetus [42]; our data suggest that it’s potential to modulate the S1P axis, and therefore trophoblast migration, should also be considered as a contributory factor.

## Conflict of interest

The authors have no conflicts of interest to declare.

## Author contributions

MW conceived ideas for the project, performed some of the experiments, analysed much of the data and wrote the paper. KA-S, SF-S, CT, EC, SB and DA all contributed experimental data and SF-S and SB critically reviewed the paper. EDJ conceived ideas for the project, contributed to experimental design, coordinated the study and edited the manuscript. All authors reviewed the results and approved the final version of the manuscript.
